# I'll take the low road: the evolutionary underpinnings of visually triggered fear

**DOI:** 10.3389/fnins.2015.00414

**Published:** 2015-10-29

**Authors:** James A. Carr

**Affiliations:** Department of Biological Sciences, Texas Tech UniversityLubbock, TX, USA

**Keywords:** stress, anxiety, fear, amphibian, fish, superior colliculus, optic tectum

## Abstract

Although there is general agreement that the central nucleus of the amygdala (CeA) is critical for triggering the neuroendocrine response to visual threats, there is uncertainty about the role of subcortical visual pathways in this process. Primates in general appear to depend less on subcortical visual pathways than other mammals. Yet, imaging studies continue to indicate a role for the superior colliculus and pulvinar nucleus in fear activation, despite disconnects in how these brain structures communicate not only with each other but with the amygdala. Studies in fish and amphibians suggest that the neuroendocrine response to visual threats has remained relatively unchanged for hundreds of millions of years, yet there are still significant data gaps with respect to how visual information is relayed to telencephalic areas homologous to the CeA, particularly in fish. In fact ray finned fishes may have evolved an entirely different mechanism for relaying visual information to the telencephalon. In part because they lack a pathway homologous to the lateral geniculate-striate cortex pathway of mammals, amphibians continue to be an excellent model for studying how stress hormones in turn modulate fear activating visual pathways. Glucocorticoids, melanocortin peptides, and CRF all appear to play some role in modulating sensorimotor processing in the optic tectum. These observations, coupled with data showing control of the hypothalamus-pituitary-adrenal axis by the superior colliculus, suggest a fear/stress/anxiety neuroendocrine circuit that begins with first order synapses in subcortical visual pathways. Thus, comparative studies shed light not only on how fear triggering visual pathways came to be, but how hormones released as a result of this activation modulate these pathways.

## Introduction

**Figure 1:**
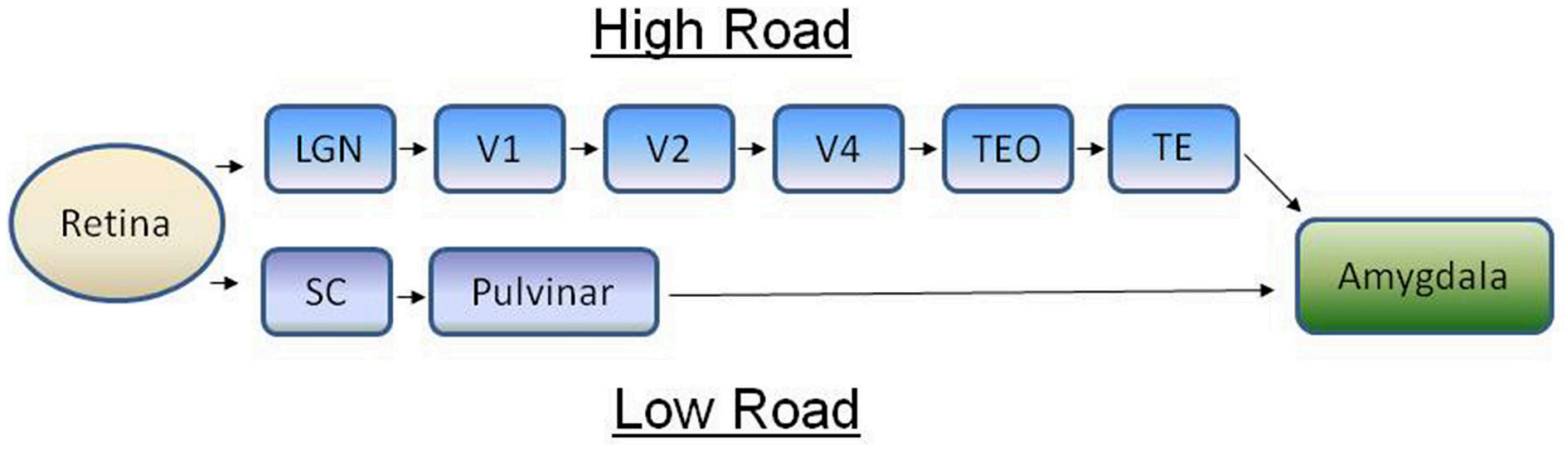
**The concept of the high road (cortical) and low road (subcortical) visual pathways for processing fear in primates**. On the high road, visual information from retinal ganglion cells is relayed to the visual cortex via the lateral geniculate nucleus, a brain area in the thalamus. Visual information is processed through several areas of the cortex before it's sent to the amygdala, whereupon autonomic and endocrine mediators of fear are engaged. On the low road, visual information is sent first to the superior colliculus in the midbrain before being relayed to the amygdala via the pulvinar nucleus. Adapted from Pessoa and Adolphs (2010). LGN, lateral geniculate nucleus; SC, superior colliculus; TE, inferior temporal cortex; TEO, inferior temporal cortex; V, visual cortex.

Since the benchmark studies of Hans Selye a tremendous amount has been learned about the endocrine physiology of stress, fear, and anxiety. As important as the adrenal glands are for coping with stressors, however, they cannot detect changes in the environment and precisely how various sensory modalities trigger the hypothalamus-pituitary-adrenal (HPA) axis is still poorly understood. Relevant to this question is a debate regarding whether cortical or subcortical visual pathways trigger fear in humans (Ohman et al., 2007; Pessoa and Adolphs, 2010). Of the several visual systems one could argue exist in the human brain, it is the lateral geniculate nucleus (LGN)-striate cortex-amygdala and the superior colliculus (SC)-pulvinar nucleus-amygdala pathways that are front and center in this debate (Figure 1), with some (Pessoa and Adolphs, 2010) questioning whether primates rely on the subcortical pathway at all to deal with visual threats. Yet, even though most vertebrates inhabiting the earth (with the possible exception of snakes, Northcutt and Butler, 1974) rely on subcortical visual pathways for detecting visual threats, the bulk of what we know about fear and the visual system comes largely from studies on humans and laboratory mammals. In part this is understandable, because it is only recently that we've begun to learn that non-mammalian vertebrates experience fear that is comprised of anatomical (Ogawa et al., 2014), behavioral (Jesuthasan, 2012), neurochemical (Silva et al., 2015), and emotive components (Kalueff et al., 2012) which are similar to fear in humans and, thus, can be distinguished independently from anxiety (Perusini and Fanselow, 2015). Now that methods have been developed for assessing fear in non-mammalian species (Kalueff et al., 2012; Ogawa et al., 2014) can we use a comparative approach to gain perspective on the importance of the SC-pulvinar-amygdala pathway for processing fear in humans? In this review I will examine the comparative and evolutionary biology of subcortical visual pathways and their role in processing fear after first reviewing the situation in humans.

## The low road, high road debate

The concept of two visual systems, one a subconscious pathway that localizes objects and the second an evolutionarily newer visual system that identifies objects, dates back to the 1960s and work in hamsters by Schneider (1967, 1969) and work in frogs by Ingle (1973). More recent imaging studies showing that the SC plays a central role in the response to a wide variety of stressors (Javanmard et al., 1999; Cornwell et al., 2012; Kessler et al., 2012; Steuwe et al., 2015), and studies showing ascending projections to the amygdala that are relayed through the pulvinar nucleus (Morris et al., 1999, 2002; Liddell et al., 2005; Ohman et al., 2007), has re-focused this debate on the importance of the SC, in particular, in processing fear. Are visual threats processed consciously or in a more reflexive, subconscious, way by the brain? This is more or less the question at the heart of the debate regarding the processing of visually triggered fear. My goal here is not review all of the arguments on both sides of the debate, as this already has been done by other authors (Ohman et al., 2007; Pessoa and Adolphs, 2010), but rather to summarize the issues at the heart of this debate before moving on to addressing the evolution of subcortical visual pathways and their connections to the hypothalamus and brainstem autonomic control areas. These issues include data gaps in precisely how, and even if, the pulvinar nucleus relays information from the SC to the amygdala, the relative response latencies of the high road and low road pathways, and the necessity of the SC for relaying visual information to the pulvinar nucleus.

### All roads lead to the amygdala

**Figure 2:**
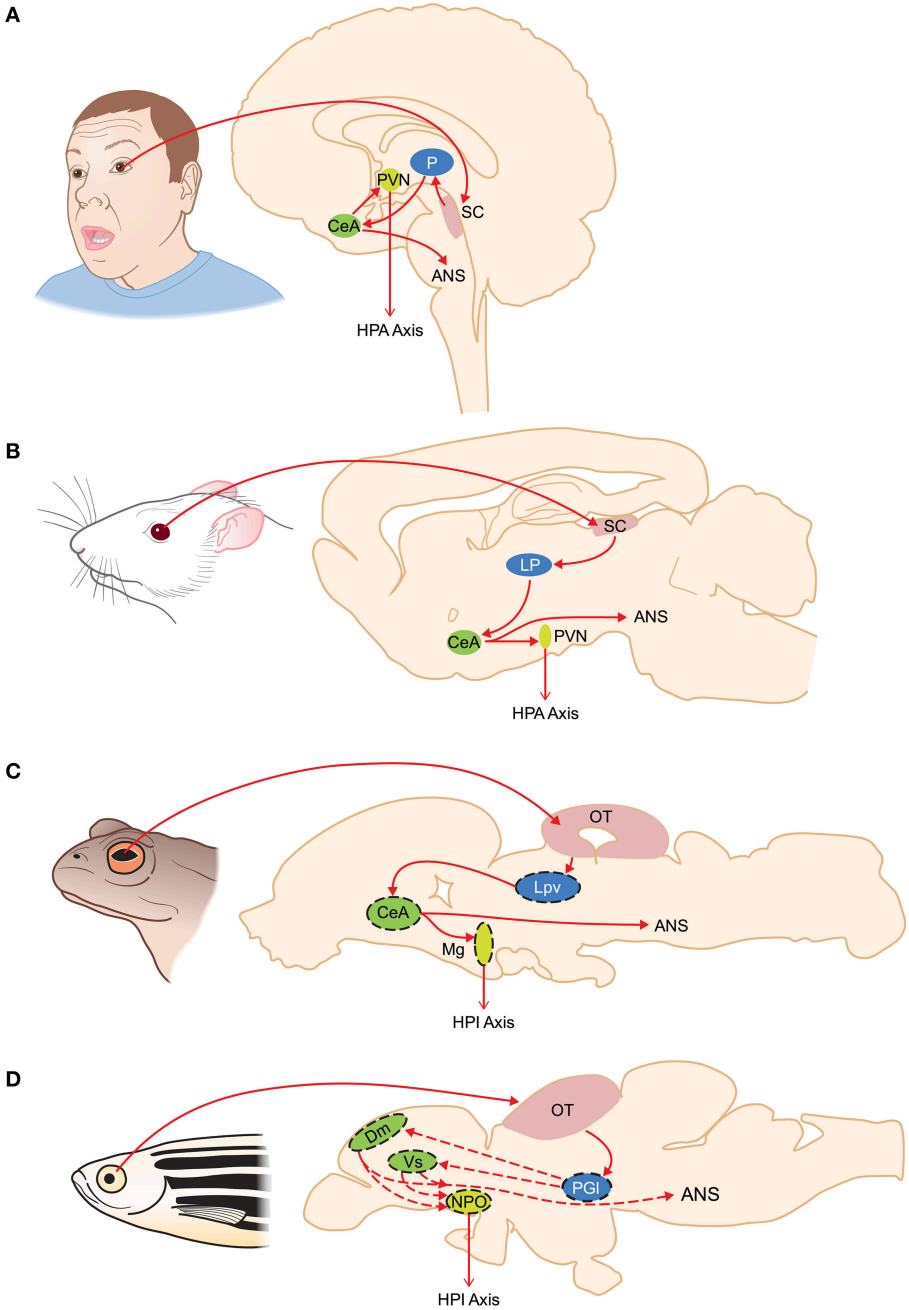
**Organization of subcortical visual pathways triggering fear in humans (A), rodents (B), amphibians (C), and fish (D)**. Homologous structures are color coded. Putative homologies are represented by dashed lines. ANS, autonomic nervous system; CeA, central amygdala; Dm, medial zone of the dorsal telencephalon; HPA, hypothalamus-pituitary-adrenal axis; HPI, hypothalamus-pituitary-interrenal axis; LP, lateroposterior dorsal thalamic complex; Lpv, lateral posteroventral thalamic nucleus; Mg, magnocellular preoptic nucleus; NPO, nucleus preopticus; OT, optic tectum; P, pulvinar nucleus; PGl, lateral preglomerular nucleus; PVN, paraventricular nucleus; SC, superior colliculus; Vs, supracommisural nucleus of the area ventralis telencephali.

Exposure to fearful visual stimuli leads to changes in electrical activity of the amygdala in humans (Morris et al., 1996) and monkeys (Leonard et al., 1985; Nakamura et al., 1992; Kuraoka and Nakamura, 2006, 2007; Gothard et al., 2007; Hoffman et al., 2007; Kuraoka et al., 2015) although the amygdala does not receive any direct retinal innervation. LeDoux (1990, 1994) suggested that visual threats were primarily conveyed to the limbic system via the SC-pulvinar pathway, the so-called “low road” (Figure 1). Although some (Pessoa and Adolphs, 2010) have argued that this terminology (with the “high road” being the LGN-striate cortex pathway) vastly over simplifies the complexity with which the pulvinar nucleus communicates with the cortex in response to a threat, this concept has been useful in highlighting the evolutionary underpinnings of fear as I will discuss further along in this review. Via a linkage with the amygdala both the SC-pulvinar and LGN-striate cortex pathways can hook up with two of the major motor outputs for fear, the HPA axis and the sympathetic nervous system. The subcortical route for processing visually triggered fear is supported by studies showing that neuronal pathways exist between the SC and pulvinar nucleus (Grieve et al., 2000; Stepniewska, 2004) and the pulvinar nucleus and amygdala (Jones and Burton, 1976; Romanski et al., 1997; Figure 2). Connections between the inferotemporal cortex, the last stop in cortical processing, and amygdala, and their role in responding to fearful stimuli, have been reviewed by others (Pessoa and Adolphs, 2010; Tamietto and de Gelder, 2010).

Because responding quickly to a threat is of paramount importance to survival, some have suggested (LeDoux, 1990; Pasley et al., 2004; reviewed by Ohman et al., 2007) that information could be carried via subcortical pathways more quickly and the increased speed in relaying information to the amygdala, in theory anyway, would be evolutionarily adaptive. Whether the low-road pathway actually improves fitness and survival has never been tested empirically, although there is considerable evidence that this pathway is important in visual threat detection (Morris et al., 1999; LeDoux, 2000; Liddell et al., 2005), especially in response to visual images of a snake (Maior et al., 2011, 2012; Le et al., 2013). Nonetheless, several observations have brought into question whether a subcortical pathway operates at all to process visual threats in humans (Pessoa and Adolphs, 2010). Pessoa and Adolphs (2010) summarize physiological data suggesting that the subcortical pathway does not process threats more quickly than the cortical pathway. In addition, SC lesions have little impact upon visual activity in the monkey pulvinar nucleus (Bender, 1983), questioning the functional connectivity between the two brain areas. While the SC projects primarily to the inferior pulvinar nucleus, the output from this area is directed to the cortex and not the amygdala (reviewed by Pessoa and Adolphs, 2010). When taken together, are these findings compelling enough to accept the hypothesis that humans, and perhaps primates in general, have lost their reliance on the SC-pulvinar-amygdala pathway to process fearful stimuli? Is this pathway truly vestigial? In contrast to other mammals, only about 20% of retinal ganglion cells (RGCs) project to the SC whereas virtually all RGCs project to the LGN in primates (Weller and Kaas, 1989), additional evidence that has led some to conclude the evolution of primate vision was accompanied by less reliance on the SC (Kaas, 2013). Along these lines it is worth mentioning that this may not be the first time in vertebrate evolution that this has happened. Examination of the evolution of the visual system in snakes suggests great reliance on retino-geniulate pathways following the degeneration of the eyes in fossorial lizards (Northcutt and Butler, 1974).

Recent evidence, including data in humans, provides some additional fuel for the low-road side of the debate, albeit without necessarily addressing the physiological and anatomical issue brought up by Pessoa and Adolphs (2010). Following up on a 2013 study demonstrating that single cells in the monkey pulvinar nucleus responded specifically to various images of snakes (Le et al., 2013), Almeida et al. (2015) provide fMRI data in humans supporting a role for the low road pathway in detecting visual images of snakes. Certainly a big question regarding the broad application of the low-road model to non-primate mammals is the degree to which the lateroposterior dorsal thalamic complex (LP) of rodents (Lent, 1982; van Groen and Wyss, 1992) acts as a thalamic relay of ascending visual information, since the pulvinar nucleus is unique to primates. In mice, evidence of a functioning SC-LP-lateral amygdala pathway for detecting visual threats in mice has just been reported (Wei et al., 2015). Within just 2 months of this report Shang et al. (2015) identified an SC-parabigeminal nucleus (PBGN)-amygdala pathway for detecting looming objects and generating fear responses in mice. The SC-PBGN-amygdala pathway is a previously undiscovered alternative to the low and high roads for initiating visually triggered fear. How such redundancy is evolutionarily adaptive remains to be seen.

### Changes in endocrine and sympathetic nervous system activity in response to a visual threat

Anatomical and functional links between the amygdalar nuclei (in particular, the central nucleus of the amygdala, CeA) and brain areas regulating the neuroendocrine stress response are well established and have been the topic of other reviews (Rodrigues et al., 2009). However, there is surprisingly little work on how activation of the SC results in neuroendocrine correlates of fear. Although the SC has long been known as the origin for tectoreticular and tectospinal neurons coordinating approach and avoidance behaviors (Huerta and Harting, 1984a,b), there are no data indicating that SC efferents innervate the rostroventral lateral medulla (RVLM) a critical premotor area involved in modulating sympathetic nervous system preganglionic cells (Gilbey and Spyer, 1993). Likewise there are no data suggesting a direct neuronal route from the SC to the paraventricular nucleus (PVN) of the hypothalamus, which houses the hypophysiotropic corticotropin-releasing factor (CRF) producing neurons regulating the HPA axis. Nonetheless, abundant electrical and chemical stimulation studies suggest that the SC, along with the inferior colliculi and the periaqueductal gray, plays a larger role as part of a midbrain defense area (Brandão et al., 2003; Pelosi et al., 2009; Iigaya et al., 2012; Dampney et al., 2013). Electrical stimulation of the SC elicits change in cardiovascular activity and SNS activity similar to that observed during panic or stress (Brandão et al., 2003). Direct optogenetic stimulation of the SC activates the HPA axis in mice (Shang et al., 2015) as does electrical stimulation of the dorsal periaqueductal gray (PAG; Lim et al., 2011), which is not surprising given the evidence for neuronal cross talk between the SC and PAG (Lim et al., 2011). Extra-amygdalar routes for SC activation of the physiological response to fearful stimuli have been demonstrated experimentally in rats (Müller-Ribeiro et al., 2014).

## The low road may be the only road in most vertebrates

In this section I will reserve my discussion to anamniotes, specifically bony fishes (Class Osteichthyes, ~28,0000 species) and amphibians (~6000 species). Collectively these groups encompass tremendous species diversity, yet a homolog of the LGN-striate cortex pathway has not been identified in either group. As such I assume that visually triggered fear, and the behavioral and neuroendocrine changes that accompany fear, are driven predominately, if not entirely, through a tectothalamic-amygdala pathway. Although it is thought that the tectothalamic pathway also predominates in reptiles and birds, birds do exhibit what is considered by some a predecessor of the LGN-striate cortex pathway, the thalamofugal pathway (Karten, 1969; Engelage and Bischof, 1993; Shimizu and Bowers, 1999) and, especially in birds with binocular vision such as owls, an area called the visual Wulst that shows striking similarities to the visual cortex (Pettigrew and Konishi, 1976a,b). For summaries of the evidence that fishes and amphibians lack a homolog of the LGN-striate cortex pathway I refer readers to articles by Mueller (2012), Laberge and Roth (2007) and Laberge et al. (2008), respectively. When referring to “homologies” I try to follow the criteria as described by Simpson (1961).

### Measuring fear in fishes and amphibians

Anxiety and fear can be thought of as belonging to the same suite of adaptive responses that are engaged in relation to the imminence of a predator encounter, with anxiety related behaviors (including wariness and vigilance) predominating prior to an encounter and fear-related behaviors (such as freezing, escape, and immobility, Ratner, 1967) emerging post-encounter (Perusini and Fanselow, 2015). Such fear related behaviors have been observed in non-mammalian species. For example, tonic immobility (TI) is generally distinguished from “freezing” as a last resort to predator avoidance (Ratner, 1967) and is thought to be diagnostic of extreme fear in humans (Abrams et al., 2009; Volchan et al., 2011). Exposure to a visual threat induces TI as well as other components of defensive behavior in fishes and amphibians (Gargaglioni et al., 2001; Verbeek et al., 2008; Toledo et al., 2010; Narayan et al., 2013). In toads (genus *Bufo*), TI is part of a characteristic last ditch effort to avoid predation by snakes (Ewert, 1980).

Zebrafish have rapidly become a model for studying the visual basis of fear, anxiety (Blaser et al., 2010; Maximino et al., 2010a,b), and emotions in general (Kalueff et al., 2012). In addition to escape, avoidance, and leaping or jumping, the preference of zebrafish for a dark surroundings and for seeking shelter have been used to assess fear and anxiety in this species (Blaser et al., 2010; Blaser and Rosemberg, 2012; Maximino et al., 2012). Zebrafish respond to the sight of a visual live predator with escape behavior and “leaping” (Bass and Gerlai, 2008), a presumably defensive behavior not usually considered part of the mammalian repertoire. Similar behaviors were observed with animated images of a series of ecologically relevant piscine predators (Ahmed et al., 2012). Interestingly, zebrafish also demonstrate fear in response to the overhead presentation of a bird silhouette (Luca and Gerlai, 2012a,b). Exposure of zebrafish to a rapidly expanding dot also elicits fear-associated behaviors (Luca and Gerlai, 2012b).

The promise of using genetically tractable zebrafish for dissecting the molecular and genetic basis of visually triggered fear has led to the use of robotic predators (Cianca et al., 2013), which ostensibly eliminate individual variation associated with using a living predator. Exposure to a robotic predator elicited the most robust avoidance behavior in zebrafish, especially compared to a computer generated visual simulation of a predator (Ladu et al., 2015a,b). Exposure to two robotic predators, an Indian leaf fish and an Indian pond heron (*Ardeola grayii*) produced fear as gauged by performance in a light/dark preference test and a shelter seeking test, respectively (Cianca et al., 2013).

Classical fear conditioning has been demonstrated in some fish species. Using a combination of social avoidance and classical conditioning in rainbow trout, fish learned in 7 days to escape a larger conspecific aggressor (unconditioned stimulus, US) when exposed to a stoppage of water flow (conditioned stimulus, CS) in the aquaria (Carpenter and Summers, 2009). There are now several reports of successful fear conditioning in zebrafish (see Aoki et al., 2013; Amo et al., 2014), although most use electrical shock, not a visual threat, as a US.

Exposure to a visual threat elevates HPA axis activity in fishes (Woodley and Peterson, 2003; Barcellos et al., 2007; Verbeek et al., 2008; Oliveira et al., 2013) and amphibians (Narayan et al., 2013) as evidenced by elevated cortisol/corticosterone (CORT) secretion. In some of these studies and, in fact on an increasing basis in the fish and amphibian literature, CORT secreted into the tank water is used as a surrogate measurement for blood CORT, which can be challenging to measure in small animals. Despite the technical challenges in using immunological based assays to measure CORT conjugates in water, the results seem qualitatively similar to those achieved through measurement of blood or whole body CORT.

One of the hallmarks of SC stimulation in mammals is activation of SNS and subsequent changes in cardiovascular activity. Cordeiro Cordeiro de Sousa and Hoffmann (1985) showed that in toads (*Bufo paracnemis*) avoidance behavior and electrical stimulation of either the caudal optic tectum (OT) or the pretectal area results in an increase in sympathetic nervous system activity and heart rate, suggesting that this aspect of visually triggered fear is qualitatively similar to that in mammals.

### Threat detection and the optic tectum (OT)

The role of the OT in detecting visual threats and coordinating appropriate avoidance responses has been studied in many non-mammalian vertebrates, although recent work in zebrafish and the work by Jerome Lettvin, Jorg-Peter Ewert, and David Ingle and colleagues in frogs and toads provides the most comprehensive look at this ancestral role for the SC. Ablation of the OT prevents virtually all behaviors related to visual avoidance in frogs (Ingle, 1973) while neurons in the thalamus appear to receive the visual information regarding threats (Ewert, 1967). Lesions placed within the visual thalamus of toads abolish avoidance behavior (Ewert, 1968). This observation, and the observation that tectally lesioned frogs still recognize stationary objects led to the concept of the two visual systems in the anuran brain (Ingle, 1973).

Given the recent findings of an SC-PBGN-amygdala pathway for detecting visual threats in mice (Shang et al., 2015), it is interesting to consider if the nucleus isthmi (NI), the likely homolog in anurans of the PBGN (Caudill et al., 2010), plays a similar role in anurans. The anuran NI is an intensely cholinergic bilateral nucleus lying ventral to the caudal part of the OT, with reciprocal ipsilateral and contralateral connections to the OT (Dudkin et al., 2007). There is compelling evidence that one major role for the NI is feature selection, that is isolating one particular visual stimulus via feedback with the OT (Gruberg et al., 2006). It's role in conveying ascending information about visual threats to the anuran amygdala is unstudied.

Not surprisingly, a role for the OT in visual threat detection has been confirmed in the zebrafish. Preuss et al. (2014) showed that different groups of retinal ganglion cell fibers innervating the OT fire in response to prey and predator size visual images. Multiphoton imaging was used by Temizer et al. (2015) to demonstrate that the OT receives afferent input from retinal ganglion cells firing in response to looming objects, and that laser ablation of retinal afferents in the OT abolishes this response. A role for the NI in the visual response to looming objects has been reported in goldfish and bluegill sunfish (Gallagher and Northmore, 2006).

### Are snake detecting cells in the amphibian thalamus homologous to cells in the primate pulvinar nucleus that respond to images of snakes?

The primate pulvinar nucleus, the critical relay in the low road hypothesis, is not found in non-primate mammals let alone frogs and fishes. Yet there is evidence that areas of the anuran caudal thalamus contain snake detector cells as recently suggested for the primate pulvinar nucleus (Le et al., 2013, 2014; Etting and Isbell, 2014). In fact, such cells were proposed to exist in the visual thalamus of the toad in the 1960s and 1970s by Ewert's group (Ewert, 1974, 1980). Ewert's work showed that visual information about predators reaches retinorecipeint areas of the lateral thalamus of toads, activating so-called “TH3” and “TH4” neurons' based on their electrophysiological properties (von Wietersheim and Ewert, 1978). Lesions of the visual thalamus in toads will cause animals to approach and snap at threatening stimuli as if they were food (Ewert, 1967, 1968), suggesting an inhibitory pathway between the lateral thalamus and OT in toads. This inhibitory pathway originates within TH3 and TH4 neurons in the lateral posterodorsal thalamic nucleus (Lpd), lateral posterior thalamic nucleus (Lp), and pretectum that project to the ipsilateral OT (Lázár, 1989). Lesioning of Lpd neurons prevents the behavioral response to fearful visual stimuli, and antidromic stimulation of the ipsilateral tectum activates neurons in the Lpd and lateral posteroventral thalamic (Lpv) nucleus (Ewert et al., 1996). Thus, the neurons in the anuran visual thalamus are important for detecting visual threats and inhibiting the OT as part of a neuronal mechanism balancing foraging/predator avoidance tradeoffs.

There is yet much to learn about putative snake detecting cells in both primates and amphibians, including the precise stimulus features causing them to fire. What we do know based on careful work in primates is that the latencies to respond to snake images are shorter in the SC than in the pulvinar nucleus (Le et al., 2014), which is consistent with a circuit that travels to the SC prior to the pulvinar nucleus. However, the typically more rapid response of the SC also has been attributed by Pessoa and Adolphs (2010), based upon data from Boehnke and Munoz (2008), to rapid eye movement associated with orienting. The pulvinar neurons responding to snakes appear to be threat sensitive, discriminating between threatening and non-threatening postures of snakes (Le et al., 2014) which also is consistent with how snake detector cells are believed to work in anurans (Ewert, 1980). It is unknown whether these cells are sensitive to snake related phobias or detect other potential fearful stimuli, such as spiders, is unclear. Furthermore, it is presently unclear whether the pulvinar cells detect snake images or are simply responding to snake detecting cells in other areas, such as the SC, with the latter possibility seemingly consistent with the response latency measurements mentioned above. Whether the visual areas of the anuran lateral thalamus are homologous to the pulvinar nucleus remains to be determined, but the idea that the pulvinar neurons responding to snakes evolved hundreds of millions of years ago is an intriguing one.

### Connections between the OT and visual thalamus

In amphibians tectal efferents travel to various thalamic nuclei, with the results obviously dependent upon the species, and the type of neuronal tracer used (Wilczyniski and Northcutt, 1977; Rettig, 1988; Montgomery and Fite, 1991; Chahoud et al., 1996; Dicke, 1999; Horowitz and Simmons, 2010). Tectal innervation of the thalamus has generally been reported to be wide spread through the dorsal and ventral thalamus across both anuran and urodele species. For example, in plethodontid salamanders tectothalamic fibers arise from at least three different tectal cell types ranging from cells having large widespread dendritic trees to neurons having small dendritic trees (Dicke, 1999). At present there are no studies examining the pattern of this innervation in concert with labeling of thalamic neurons ascending to the CeA.

In contrast to the situation in amphibians, and despite the fact that the use of zebrafish as a model for fear conditioning has accelerated rapidly, there is uncertainty about the thalamic relay for visually triggered fear in fishes. Part of the issue in identifying homologies to the low road circuitry in tetrapods is the fact that the fish forebrain undergoes a process of eversion during development rather than invagination, as is the case in tetrapods (see Demski, 2003, for a summary). And while there is no question that fish species are excellent models for behavioral studies on predator avoidance and fear, there is little evidence for a tectothalamic-amygdala pathway in fish that is developmentally, hodologically, or even functionally similar to the SC-pulvinar-amygdala pathway in primates (Northcutt, 2006; Mueller, 2012). There is evidence that the preglomerular nucleus (PG, which forms near the posterior tubercle and is unique to ray-finned fishes) may fill the role of a visual relay between the OT and telencephalic areas including the amygdala (Northcutt, 2006, 2008; Yamamoto and Ito, 2008). In teleosts the lateral preglomerular nucleus (PGl) receives tectal afferents and projects to the telencephalon. In cladisteans and chondrosteans the role of visual relay is carried out by the nucleus medianus of posterior tubercle (Northcutt et al., 2004), which is absent in teleosts (Northcutt, 2008).

Unfortunately there is still uncertainty also about whether the PG complex (teleosts) or nucleus medianus of posterior tubercle (cladisteans, chondrosteans) are homologous to the dorsal thalamus of tetrapods. Examination of the PG complex in species basal to the actinoptergyii phylogenetic lineage (polypterus for example) reveal that the posterior tubercle, from which the preglomerular complex arises during development, is relatively unmigrated and simple in adult specimens (Bradford and Northcutt, 1983; Northcutt, 2008). The PG complex first becomes recognizable in sturgeon. Developmental studies however paint a different picture, as paired box 6 (pax6)- or distal-less homeobox 2 (dlx2)-positive cells apparently migrate to the preglomerular complex in medaka (Ishikawa et al., 2007) from the alar plate of the diencephalon. Thus, whether the low road has always traveled through the equivalent of the dorsal thalamus in vertebrates or arose as a result of convergent evolution in tetrapods and ray finned fishes remains unsettled.

In addition to the PGl, rostral areas of the preglomerular complex as well as the prethalamicus and VMT (ventromedial nucleus of the thalamus) have been proposed to act as relays for ascending visual information in fishes (Demski, 2003). There also is some evidence that the dorsoposterior thalamic nucleus (DP) may act to relay visual information to the pallial homologs of the amygdala in fish, but there are equivocal findings regarding its connection to the OT (summarized by Mueller, 2012). Early studies (Sharma, 1975; Luiten, 1981) in goldfish failed to show tectal afferents innervating the DP while such pathways have been observed in catfish (Striedter, 1990).

### The role of the amygdala in fishes and amphibians

Amphibians are generally believed to have a pallial homolog of the tetrapod CeA based on neurochemical architecture (somatostatin, neuropeptide Y, tyrosine hydroxylase, and nitric oxide synthase as markers) and tracing studies (Moreno and González, 2005, 2006, 2007; Mühlenbrock-Lenter et al., 2005). Numerous cells in the dorsal thalamus project to the anuran CeA (Moreno and González, 2005), with the most numerous cells being located in caudal regions of the lateral thalamic nucleus and Lpv. Efferent projections from the amphibian CeA travel to endocrine (hypothalamus, preoptic area) and autonomic (locus coeruleus, nucleus of the solitary tract, and spinal cord) systems (Moreno and González, 2005). The visual thalamus in amphibians receives retinal afferents at three locations, and while neurons in the dorsal thalamus that project to the amygdala respond to optic nerve stimulation, this response is inhibitory. Furthermore, these neurons do not have dendritic fields directly in contact with retinal afferents and their response to optic nerve stimulation is inhibition (Roth et al., 2003). Thus, assuming that glutamate is the principal neurotransmitter in retinal afferents, and that as such retinal signals are primarily excitatory, then retinal information arrives at neurons in the dorsal thalamus after having already been filtered through multiple synapses (Roth et al., 2003). Furthermore, this innervation is inhibitory, and may involve GABAergic projections from the ventral thalamus as work in the urodele *Plethodon jordani* suggests (Roth and Grunwald, 2000).

Not only is a homolog of the tetrapod extrageniculate thalamic relay for ascending visual information disputed for fishes (see above), but there is some question as to whether a functional homolog of the autonomic amygdala (CeA) even exists. Mueller (2012) identifies the medial zone of the dorsal telencephalon (Dm) as a pallial area homologus to the amygdala, although does not elaborate on which areas of the Dm might be considered the “autonomic amygdala.” Northcutt (2006) reported that several subdivisions of the PG, including those receiving visual inputs from the OT, project to the Dm in goldfish. In a recent review by Maximino et al. (2013), however, the authors relegate the Dm to status as a homolog of the basolateral amygdala and suggest that subpallial areas, specifically the supracommisural nucleus of the area ventralis telencephali (Vs), is the homolog of the CeA. Indeed molecular marker analysis suggests that the Vs shares the most homology with the mammalian CeA (Alderman and Bernier, 2007; Ganz et al., 2012). Markers for the CeA found in the Vs include CRF (Pepels et al., 2002; Alderman and Bernier, 2007) and GAD67, a biosynthetic enzyme in the GABA synthesis pathway. Whether, the Vs receives inputs from the preglomerular complex or projects to brainstem autonomic nuclei and the hypothalamus is untested. Interestingly, the lobe finned fishes (sarcopterygii) appear to have a pallial homolog of the tetrapod CeA (Gonzalez and Northcutt, 2011; Northcutt and González, 2011).

So what we have to date are isolated examples of pallial and posterior tubercular cell groups that may or may not be homologous to the CeA and extrageniculate relays in the dorsal thalamus of tetraprods, respectively. There doesn't appear to have been any attempts so far to link the PGl outputs to CeA related areas of the pallium, in fact, most evidence suggests that projection neurons in the PGl target the Dm and Dl. In light of the difficulties in identifying precisely how ascending visual information reaches parts of the pallium that regulate the hypothalamus and autonomic areas during visually triggered fear, what is truly fascinating is that both fishes and amphibians exhibit endocrine responses to visual threats that are recognizably similar to those generated by the low road pathway in mammals. Moreover, there is clear evidence that the OT is central to mediating the sensorimotor decisions that must take place when challenged by a visual threat (Ingle, 1973; Preuss et al., 2014; Temizer et al., 2015).

## Stress hormone modulation of subcortical visual pathways

**Figure 3:**
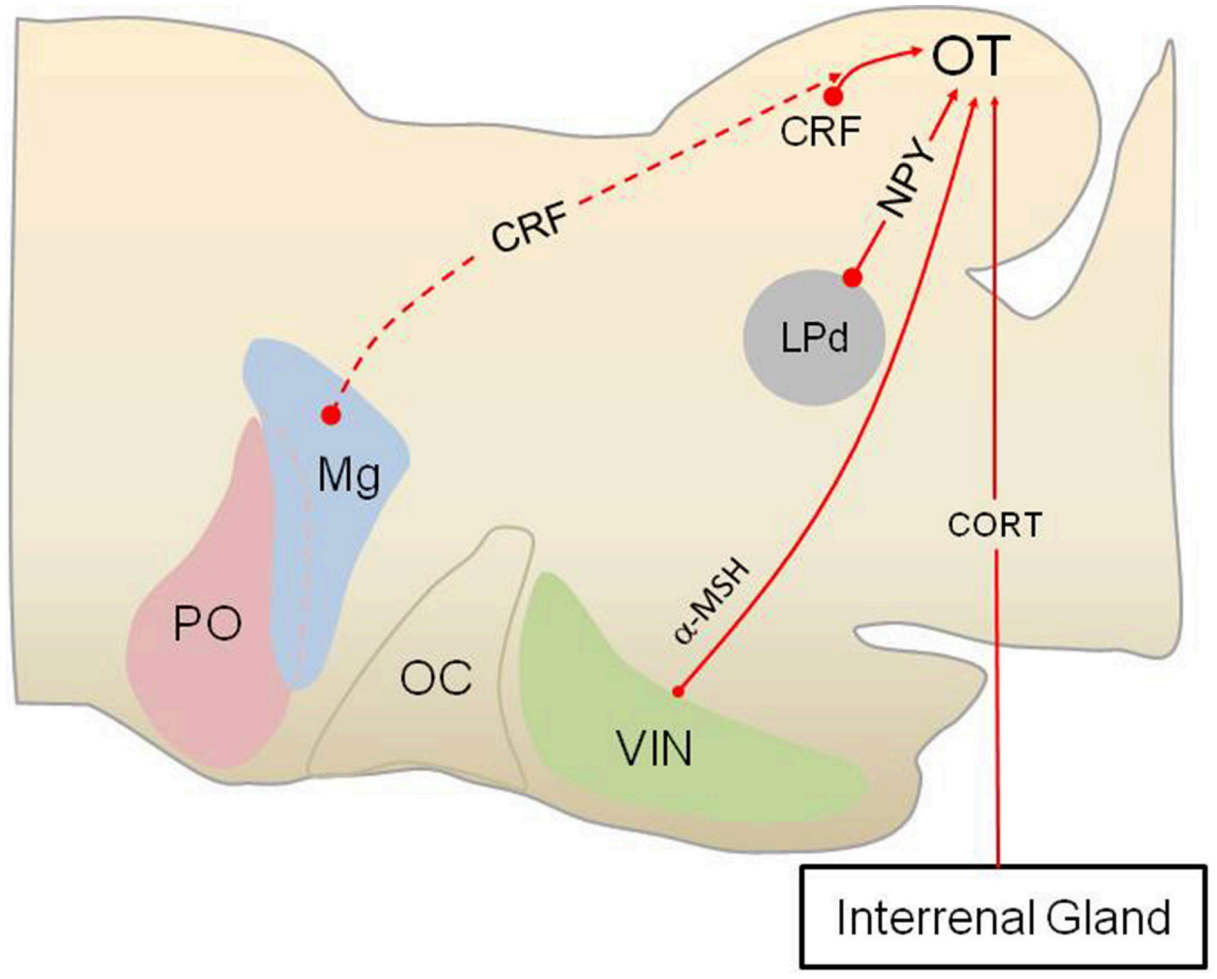
**Stress hormones and neuropeptides modulate subcortical visual pathways in anurans**. Anurans provide an excellent model for studying how various stress hormones modulate subcortical visual pathway, as the lateral geniculate-striate cortex pathway is absent in amphibians as a group. As shown in this sagittal section of the anuran brain (rostral to the left), corticotropin-releasing factor (CRF) is produced by interneurons in the optic tectum (OT), in addition hypophysiotropic CRF neurons (dashed red line, indicates possible innervation) may project to the retinorecipient layers (layer 9) of the OT. N-acetyl ACTH_1-13_ amide (alpha melanocyte-stimulating hormone, α-MSH) neurons in the ventral infundibular nucleus (VIN), homologous to the arcuate nucleus in mammals, also projects to the OT and modulate prey capture. Neuropeptide Y (NPY) produced by neurons in the lateroposterodorsal nucleus (LPd) of the thalamus acts within the OT to reduce approach and prey capture behavior, presumably when a visual threat is present, as this part of the thalamus receives visual information about predators. Corticosterone (CORT) produced by cells in the interrenal glands acts on glucocorticoid receptors in the OT to modulate subcortical visual processing in an as of yet unidentified way. PO, preoptic area; Mg, magnocellular preoptic nucleus; OC, optic chiasm.

Although it is already established that subcortical visual pathways communicate with the HPA/HPI axes, there also is evidence for a reciprocal role for HPA axis neuropeptides and hormones in regulating the SC/OT (Figure 3). Receptors for CORT (glucocorticoid receptors, GRs) are found in the tecta of fishes (Teitsma et al., 1998), amphibians (Yao et al., 2008), and birds (Wiggert and Chader, 1975; Shahbazi et al., 2011), suggesting an evolutionarily ancient role for CORT in regulating some as of yet unknown aspect of sensorimotor processing in this brain area. In rainbow trout (*Oncorhynchus mykiss*) GRs are concentrated in the periventricular layer of the OT (Teitsma et al., 1998). GR-positive cells are located in several layers of the African Clawed frog (*X. laevis*) OT (Yao et al., 2008). Exposure to a stressor that elevates CORT secretion enhanced the immunoreactive staining for the Krüppel-like factor 9 (KLF9) in the OT of *X. laevis*, an effect that was blocked by prior treatment with the glucocorticoid antagonist mifepristone (RU486) and replicated with administration of exogenous CORT (Bonett et al., 2009).

One interesting question that is currently unresolved is whether CORT targets the SC/OT and perhaps dPAG as part of a feedback loop for regulating stress. Recent data in mice indicate that selectively stimulating cells in the SC leads to elevated CORT secretion (Shang et al., 2015) while work in rats has shown that stimulating the dPAG also activates the HPA axis (Lim et al., 2011). Perhaps one role for GRs in this midbrain defense area is to shut off the response to visual threats (Figure 3).

Moderate to high concentrations of CRF have been observed in the OT of several amphibian and fish species (reviewed by Carr et al., 2010). In African clawed frogs, *in vitro* release of CRF is greater from the OT than any other brain area (Carr et al., 2013). CRF-ir neurons are located in tectal layers 6 and 8 (Carr et al., 2010), areas known to possess interneurons with dendrites extending to layer 9 in order to intercept incoming information from the retina and pretectal areas and visual thalamus. Peripheral or central administration of the peptide reduces visually-guided prey capture (Carr et al., 2002; Crespi and Denver, 2004; Morimoto et al., 2011), but whether this is a direct effect on tectal CRF receptors is currently being tested in my laboratory. Exposure to a reactive stressor (ether vapors) elevates the tectal content of CRF while food deprivation lowers tectal CRF (Prater et al., 2014), suggesting a possible role in managing threat avoidance/prey capture decision making.

Receptors that mediate the action of various melanocortin peptides (except ACTH) have been observed in the OT. In rats, retinal ganglion cells express MC3R, MC4R, and MC5R receptors with MC4R being the most abundant (Lindqvist et al., 2003). Expression of the receptors and the POMC mRNA is reduced in the SC after transection of the optic nerve (Lindqvist et al., 2003). In toads (*Spea multiplicata* and *Bufo speciosus*, detectable levels of N-acetyl ACTH_1-13_ amide, or α-melanocyte-stimulating hormone (α-MSH), were found in the OT by radioimmunoassay and immunocytochemistry (Olsen et al., 1999; Venkatesan and Carr, 2001). Proopiomelanocortin (POMC) -producing cells in the non-mammalian brain are generally limited to one or two hypothalamic areas, depending upon the species, with every species examined to date possessing POMC cells in the ventral infundibular nucleus (presumably homologous to the arcuate POMC cell group in mammals; Venkatesan and Carr, 2001). Thus, the α-MSH-ir fibers found in the OT arise probably from the hypothalamus. Peripheral administration of α-MSH enters the brain (Olsen et al., 1999) and accelerates habituation to artificial prey in the Texas toad *Bufo speciosus* and the Great Plains toad *Bufo cognatus* (Carpenter and Carr, 1996; Olsen et al., 1999). Exposure to a non-specific stressor alters the content of α-MSH in the OT (Venkatesan and Carr, 2001), suggesting that this peptide may reduce prey-capture when a threat is present.

Prey animals, including most fish and amphibian species, are under intense selection to detect and respond appropriately to avoid or escape predators. Studies across several taxonomic groups have shown that under high predation risk situations, prey increase vigilance by reducing their foraging effort (Ferrari et al., 2009). These behavioral decisions increase immediate survival at the expense of decreased intake of food which may impose long-term costs on the individual's growth rate or reproductive output (Werner and Anholt, 1993; Lima, 1998; Cresswell, 2008). One adaptive feature of the subcortical visual circuitry, at least as it operates in amphibians, is that visual threats can shut off OT circuitry regulating approach and prey capture (Ewert, 1980). As described in detail above, retinoreceipient neurons in the thalamus process predator images and then relay this to the OT to shut down approach and foraging, even when food and a predator are present simultaneously (Ewert, 1980; Shoukfeh et al., 2003). Several pieces of evidence suggest that the thalamic neurons at the heart of this predator avoidance/foraging trade-off produce NPY. NPY-ir cells in the lateral thalamus project to retinorecipient layer 9 of the OT (Kozicz and Lázár, 1994, 2001; Chapman and Debski, 1995). Exogenous NPY reduces field potential activity in the OT in response to electrical stimulation of the contralateral optic nerve or changes in ambient light and prevents [^14^C]2-deoxyglucose, uptake in the OT in response to prey presentation (Schwippert and Ewert, 1995; Schwippert et al., 1998; Funke and Ewert, 2006). These data suggest that, in contrast to its orexigenic role in the hypothalamus, NPY inhibits either the recognition and/or the approach to prey when a predator is present.

## Summary

The subcortical visual pathway, the so-called low road for processing fear in humans, has been the predominate visual system for processing fear in vertebrates for hundreds of millions of years and across thousands of taxa. Recent studies emphasize the continuing importance of this pathway in non-primate (Shang et al., 2015; Wei et al., 2015) and primate (Almeida et al., 2015) mammals. Given the emerging role for the primate SC-pulvinar pathway in detecting visual features of snakes (Maior et al., 2011, 2012; Le et al., 2013; Almeida et al., 2015) and evidence that snakes are ecologically relevant predators (Etting et al., 2014; Shibasaki et al., 2014), it is unlikely that primates as a group have abandoned the low road altogether for processing fear, despite existing data gaps in our understanding of SC-pulvinar-amygdala connectivity (Pessoa and Adolphs, 2010) and observations that only about 20% of retinal afferents travel to the SC in primates (Weller and Kaas, 1989). Obviously more work is needed on the connectivity of the SC and pulvinar nucleus with respect to processing visual threats and connectivity of the SC with downstream mediators, specifically the sympathetic nervous system and HPA axis. The concepts of the low road/high road/many roads for processing of visual threats must also accommodate new data for a newly discovered subcortical visual pathway traveling from the SC to the PBGN and then to the amygdala.

We can say with certainty that the neuroendocrine response to visual threats has remained relatively unchanged for hundreds of millions of years. However, the pathways conveying information about threats to the HPA/HPI axes and sympathetic nervous system may have evolved more than once. It is generally believed that the anamniote OT is homologous to the SC. There seems to be good agreement that amphibians possess a well-developed autonomic amygdala that may be homologous with the CeA and that receives afferents from the lateral posterior thalamus. In ray finned fishes the best candidate for the relay of ascending visual information seems to be the PGl, but the PG complex is poorly if at all developed in basal actinopterygians. There also is considerable uncertainty regarding the arrangement of the autonomic amygdala in the forebrain of ray finned fishes. Interestingly, lungfish seem to have an arrangement that most resembles amphibians, lending more support to the idea that the PGl-telencephalic pathway is not an ancestral tetrapod trait. Is the PGl of thalamic origin? If not, are there extra-thalamic visual relays yet to be discovered in humans? Do basal actinopterygians have a portion of their thalamus dedicated to visual relay functions as in tetrapods? Are human and primate snake “detecting” cells a relatively recent evolutionary acquisition or are they homologous to snake detecting cells in the visual thalamus of anurans? These are some of the questions raised in this review that await further research. In particular, identifying when the tectothalamic-amygdala pathway first evolved seems of critical importance.

Although, large datasets support a role for the SC/OT-thalamus-amygdala pathway for processing the response to fearful stimuli in tetrapods and fish, more direct linkages between the SC/OT and the endocrine and autonomic pathways activated during fear must be considered. Do descending efferents from the SC/OT target cells in the RLVM, a critical brain area for pre-motor control of the sympathetic nervous system? Do tectospinal pathways also target preganglionic cells of the sympathetic nervous system located in the intermediolateral (IML) cell column of the spinal cord? What pathways mediate the regulation of the HPA axis by SC/OT cells? Is this control mediated through the thalamus-amygdala link or are there more direct routes between the SC/OT and the PVN (and its homologs)?

Anurans in particular have turned out to be important neuroethological models for examining how stress hormones and other neuropeptides act to modulate the OT when threats are present. These studies emphasize the role that the OT plays as part of complete neuroendocrine circuit that begins not in the hypothalamus, but at the first order synapses receiving visual information in the OT. Thus, the OT not only triggers activity of the HPA axis in response to visual threats but is in turn modulated by these and other hormones released during stress. Determining whether stress hormone modulation of the OT plays a role in stress-enhanced fear learning (SEFL; Rau et al., 2005) would seem to be an important goal for future research, as least as SEFL applies to visually triggered fear. Understanding precisely how homeostatic and stress bioregulators modulate the recognition of and response to threats is an exciting area for future research.

Finally, our understanding of fear and anxiety based illness in humans has much to gain from the study of fear and anxiety in anamniotes. The fact that anxiety, fear, and fear-learning can now be studied in species using only the low road pathway may offer tremendous insight into the significance and role of this pathway in humans.

### Conflict of interest statement

The author declares that the research was conducted in the absence of any commercial or financial relationships that could be construed as a potential conflict of interest.

## References

[B1] Abrams M. P. Carleton R. N. Taylor S. Asmundson G. J. G. (2009). Human tonic immobility: measurement and correlates. Depress. Anxiety 26, 550–556. 10.1002/da.20462 19170102

[B2] Ahmed T. S. Fernandes Y. Gerlai R. (2012). Effects of animated images of sympatric predators and abstract shapes on fear responses in zebrafish. Behaviour 149, 1125–1153. 10.1163/1568539X-00003011

[B3] Alderman S. L. Bernier N. J. (2007). Localization of corticotropin-releasing factor, urotensin I, and CRF-binding protein gene expression in the brain of the zebrafish, *Danio rerio*. J. Comp. Neurol. 502, 783–793. 10.1002/cne.21332 17436299

[B4] Almeida I. Soares S. C. Castelo-Branco M. (2015). The distinct role of the amygdala, superior colliculus and pulvinar in processing of central and peripheral snakes. PLoS ONE 10:e0129949. 10.1371/journal.pone.0129949 26075614 PMC4467980

[B5] Amo R. Fredes F. Kinoshita M. Aoki R. Aizawa H. Agetsuma M.. (2014). The habenulo-raphe serotonergic circuit encodes an aversive expectation value essential for adaptive active avoidance of danger. Neuron 84, 1034–1048. 10.1016/j.neuron.2014.10.035 25467985

[B6] Aoki T. Kinoshita M. Aoki R. Agetsuma M. Aizawa H. Yamazaki M.. (2013). Imaging of neural ensemble for the retrieval of a learned behavioral program. Neuron 78, 881–894. 10.1016/j.neuron.2013.04.009 23684786

[B7] Barcellos L. J. G. Ritter F. Kreutz L. C. Quevedo R. M. Da Silva L. B. Bedin A. C. (2007). Whole-body cortisol increases after direct and visual contact with a predator in zebrafish, *Danio rerio*. Aquaculture 272, 774–778. 10.1016/j.aquaculture.2007.09.002

[B8] Bass S. L. S. Gerlai R. (2008). Zebrafish (*Danio rerio*) responds differentially to stimulus fish: the effects of sympatric and allopatric predators and harmless fish. Behav. Brain Res. 186, 107–117. 10.1016/j.bbr.2007.07.037 17854920

[B9] Bender D. B. (1983). Visual activation of neurons in the primate pulvinar depends on cortex but not colliculus. Brain Res. 279, 258–261. 10.1016/0006-8993(83)90188-9 6640346

[B10] Blaser R. E. Chadwick L. McGinnis G. C. (2010). Behavioral measures of anxiety in zebrafish (*Danio rerio*). Behav. Brain Res. 208, 56–62. 10.1016/j.bbr.2009.11.009 19896505

[B11] Blaser R. E. Rosemberg D. B. (2012). Measures of anxiety in zebrafish (*Danio rerio*): dissociation of black/white preference and novel tank test. PLoS ONE 7:e36931. 10.1371/journal.pone.0036931 22615849 PMC3355173

[B12] Boehnke S. E. Munoz D. P. (2008). On the importance of the transient visual response in the superior colliculus. Curr. Opin. Neurobiol. 18, 544–551. 10.1016/j.conb.2008.11.004 19059772

[B13] Bonett R. M. Hu F. Bagamasbad P. Denver R. J. (2009). Stressor and glucocorticoid-dependent induction of the immediate early gene kruppel-like factor 9: implications for neural development and plasticity. Endocrinology 150, 1757–1765. 10.1210/en.2008-1441 19036875 PMC2659263

[B14] Bradford M. R. Jr. Northcutt R. G. (1983). Organization of the diencephalon and pretectum of the ray-finned fishes, Fish Neurobiology, Vol. 2, *Higher Brain Areas and Functions*, eds Davis R. E. Northcutt R. G. (Ann Arbor, MI: University of Michigan Press), 117–163.

[B15] Brandão M. L. Troncoso A. C. de Souza Silva M. A. Huston J. P. (2003). The relevance of neuronal substrates of defense in the midbrain tectum to anxiety and stress: empirical and conceptual considerations. Eur. J. Pharmacol. 463, 225–233. 10.1016/S0014-2999(03)01284-6 12600713

[B16] Carpenter A. M. Carr J. A. (1996). The effects of melanocortin peptides and corticosterone on habituation in the Great Plains toad, *Bufo cognatus*. Horm. Behav. 30, 236–243. 10.1006/hbeh.1996.0028 8918679

[B17] Carpenter R. E. Summers C. H. (2009). Learning strategies during fear conditioning. Neurobiol. Learn. Mem. 91, 415–423. 10.1016/j.nlm.2009.01.009 19340951 PMC2762607

[B18] Carr J. A. Brown C. L. Mansouri R. Venkatesan S. (2002). Neuropeptides and prey-catching behavior in toads. Rev. Comp. Biochem. Physiol. B Biochem. Mol. Biol. 132, 151–162. 10.1016/S1096-4959(01)00545-0 11997218

[B19] Carr J. A. Lustgarten J. Ahmed N. Bergfeld N. Bulin S. E. Shoukfeh O. (2010). The organization of CRF neuronal pathways in toads: evidence that retinal afferents do not contribute significantly to tectal CRF content. Brain Behav. Evol. 76, 71–86. 10.1159/000319555 20926857

[B20] Carr J. A. Zhang B. Li W. J. Gao M. M. Garcia C. Lustgarten J.. (2013). An intrinsic CRF signaling system within the optic tectum. Gen. Comp. Endocrinol. 188, 204–211. 10.1016/j.ygcen.2013.03.020 23583471

[B21] Caudill M. S. Eggebrecht A. T. Gruberg E. R. Wessel R. (2010). Electrophysiological properties of isthmic neurons in frogs revealed by *in vitro* and *in vivo* studies. J. Comp. Physiol. A Neuroethol. Sens. Neural Behav. Physiol. 196, 249–262. 10.1007/s00359-010-0511-y 20179943 PMC2860605

[B22] Chahoud B. H. Cordier-Picouet M. J. Clairambault P. (1996). Larval development of tectal efferents and afferents in *Xenopus laevis* (Amphibia anura). J. Hirnforsch. 37, 519–535. 8982811

[B23] Chapman A. M. Debski E. A. (1995). Neuropeptide-Y immunoreactivity of a projection from the lateral thalamic nucleus to the optic tectum of the leopard frog. Vis. Neurosci. 12, 1–9. 10.1017/S0952523800007264 7718491

[B24] Cianca V. Bartolini T. Porfiri M. Macrì S. (2013). A robotics-based behavioral paradigm to measure anxiety-related responses in zebrafish. PLoS ONE 8:e69661. 10.1371/journal.pone.0069661 23922773 PMC3726767

[B25] Cornwell B. R. Mueller S. C. Kaplan R. Grillon C. Ernst M. (2012). Anxiety, a benefit and detriment to cognition: behavioral and magnetoencephalographic evidence from a mixed-saccade task. Brain Cogn. 78, 257–267. 10.1016/j.bandc.2012.01.002 22289426 PMC3448553

[B26] Crespi E. J. Denver R. J. (2004). Ontogeny of corticotropin-releasing factor effects on locomotion and foraging in the Western spadefoot toad (*Spea hammondii*). Horm. Behav. 46, 399–410. 10.1016/j.yhbeh.2004.03.011 15465525

[B27] Cresswell W. (2008). Non-lethal effects of predation in birds. Ibis 150, 3–17. 10.1111/j.1474-919X.2007.00793.x

[B28] Dampney R. A. L. Furlong T. M. Horiuchi J. Iigaya K. (2013). Role of dorsolateral periaqueductal grey in the coordinated regulation of cardiovascular and respiratory function. Auton. Neurosci. Basic Clin. 175, 17–25. 10.1016/j.autneu.2012.12.008 23336968

[B29] Demski L. S. (2003). In a fish's mind's eye: the visual pallium of teleosts, in Sensory Processing in Aquatic Environments, eds Collins S. P. Marshall N. J. (New York, NY: Springer), 404–419. 10.1007/978-0-387-22628-6_21

[B30] Cordeiro de Sousa M. B. Hoffmann A. (1985). Autonomic adjustments during avoidance and orienting responses induced by electrical-stimulation of the central nervous-system in toads (*Bufo paracnemis*). J. Comp. Physiol. B Biochem. Syst. Environ. Physiol. 155, 381–386. 10.1007/BF00687482 3837021

[B31] Dicke U. (1999). Morphology, axonal projection pattern, and response types of tectal neurons in plethodontid salamanders. I: tracer study of projection neurons and their pathways. J. Comp. Neurol. 404, 473–488. 9987992 10.1002/(sici)1096-9861(19990222)404:4<473::aid-cne5>3.0.co;2-m

[B32] Dudkin E. A. Sheffield J. B. Gruberg E. R. (2007). Combining visual information from the two eyes: the relationship between isthmotectal cells that project to ipsilateral and to contralateral optic tectum using fluorescent retrograde labels in the frog, *Rana pipiens*. J. Comp. Neurol. 502, 38–54. 10.1002/cne.21308 17335048

[B33] Engelage J. Bischof H.-J. (1993). The organization of the tectofugal pathway in birds: a comparative review, in Vision, Brain, and Behavior in Birds, eds Zeigler H. P. Bischof H.-J. (Cambridge, MA: MIT Press), 137–158.

[B34] Etting S. F. Isbell L. A. (2014). Rhesus macaques (*Macaca mulatta*) use posture to assess level of threat from snakes. Ethology 120, 1177–1184. 10.1111/eth.12293

[B35] Etting S. F. Isbell L. A. Grote M. N. (2014). Factors increasing snake detection and perceived threat in captive rhesus macaques (*Macaca mulatta*). Am. J. Primatol. 76, 135–145. 10.1002/ajp.22216 24395649

[B36] Ewert J. P. (1974). The neural basis of visually guided behavior. Sci. Am. 230, 34–42. 10.1038/scientificamerican0374-34 4204830

[B37] Ewert J. P. (1980). Neuroethology an Introduction to the Neurophysiological Fundamentals of Behavior. Berlin: Springer-Verlag.

[B38] Ewert J. P. (1967). Untersuchungen über die anteile zentralnervöser aktionen an der taxisspezifischen ermüdung der erdkrüte (*Bufo bufo* L.). Z. Vergl. Physiol. 57, 263–298. 10.1007/BF00303000

[B39] Ewert J. P. (1968). Der einflußvon zwischenhirndefekten auf die visuomotorik im beute- und fluchtverhalten der erdkröte (*Bufo bufo* L.). Z. Vergl. Physiol. 61, 41–70.

[B40] Ewert J. P. Schürg-Pfeiffer E. Schwippert W. W. (1996). Influence of pretectal lesions on tectal responses to visual stimulation in anurans: field potential, single neuron and behavior analyses. Acta Biol. Hung. 47, 89–111. 9124015

[B41] Ferrari M. C. O. Sih A. Chivers D. P. (2009). The paradox of risk allocation: a review and prospectus. Anim. Behav. 78, 579–585. 10.1016/j.anbehav.2009.05.034

[B42] Funke S. Ewert J. P. (2006). Neuropeptide Y suppresses glucose utilization in the dorsal optic tectum towards visual stimulation in the toad *Bombina orientalis*: a C-14 2DG study. Neurosci. Lett. 392, 43–46. 10.1016/j.neulet.2005.09.016 16209904

[B43] Gallagher S. P. Northmore D. P. M. (2006). Responses of the teleostean nucleus isthmi to looming objects and other moving stimuli. Vis. Neurosci. 23, 209–219. 10.1017/S0952523806232061 16638173

[B44] Ganz J. Kaslin J. Freudenreich D. Machate A. Geffarth M. Brand M. (2012). Subdivisions of the adult zebrafish subpallium by molecular marker analysis. J. Comp. Neurol. 520, 633–655. 10.1002/cne.22757 21858823

[B45] Gargaglioni L. H. Pereira A. S. F. Hoffmann A. (2001). Basal midbrain modulation of tonic immobility in the toad *Bufo paracnemis*. Physiol. Behav. 72, 297–303. 10.1016/S0031-9384(00)00433-9 11274670

[B46] Gilbey M. P. Spyer K. M. (1993). Essential organization of the sympathetic nervous-system. Baillieres Clin. Endocrinol. Metab. 7, 259–278. 10.1016/S0950-351X(05)80177-6 8098208

[B47] Gonzalez A. Northcutt R. G. (2011). Functional morphology of the brains of sarcopterygian fishes: lungfishes and latimeria, in Encyclopedia f Fish Physiology: From Genome to Environment, Vol. 1–3, ed Farrell A. P. (San Diego, CA: Elsevier), 46–55.

[B48] Gothard K. M. Battaglia F. P. Erickson C. A. Spitler K. M. Amaral D. G. (2007). Neural responses to facial expression and face identity in the monkey amygdala. J. Neurophysiol. 97, 1671–1683. 10.1152/jn.00714.2006 17093126

[B49] Grieve K. L. Acuña C. Cudeiro J. (2000). The primate pulvinar nuclei: vision and action. Trends Neurosci. 23, 35–39. 10.1016/S0166-2236(99)01482-4 10631787

[B50] Gruberg E. Dudkin E. Wang Y. Marín G. Salas C. Sentis E.. (2006). Influencing and interpreting visual input: the role of a visual feedback system. J. Neurosci. 26, 10368–10371. 10.1523/JNEUROSCI.3288-06.2006 17035519 PMC6674696

[B51] Hoffman K. L. Gothard K. M. Schmid M. C. Logothetis N. K. (2007). Facial-expression and gaze-selective responses in the monkey amygdala. Curr. Biol. 17, 766–772. 10.1016/j.cub.2007.03.040 17412586

[B52] Horowitz S. S. Simmons A. M. (2010). Development of tectal connectivity across metamorphosis in the bullfrog (*Rana catesbeiana*). Brain Behav. Evol. 76, 226–247. 10.1159/000322550 21266803 PMC3202948

[B53] Huerta M. F. Harting J. K. (1984a). Connectional organization of the superior colliculus. Trends Neurosci. 7, 286–289. 10.1016/S0166-2236(84)80197-6

[B54] Huerta M. F. Harting J. K. (1984b). The mammalian superior colliculus: studies of the morphology and connections, in Comparative Neurology of the Optic Tectum, ed Vanegas H. (New York, NY: Plenum Press), 687–773. 10.1007/978-1-4899-5376-6_18

[B55] Iigaya K. Müller-Ribeiro F. C. D. Horiuchi J. Mcdowall L. M. Nalivaiko E. Fontes M. A. P.. (2012). Synchronized activation of sympathetic vasomotor, cardiac, and respiratory outputs by neurons in the midbrain colliculi. Am. J. Physiol. Regul. Integr. Comp. Physiol. 303, R599–R610. 10.1152/ajpregu.00205.2012 22814668

[B56] Ingle D. (1973). 2 visual systems in frog. Science 181, 1053–1055. 10.1126/science.181.4104.1053 4542178

[B57] Ishikawa Y. Yamamoto N. Yoshimoto M. Yasuda T. Maruyama K. Kage T.. (2007). Developmental origin of diencephalic sensory relay nuclei in teleosts. Brain Behav. Evol. 69, 87–95. 10.1159/000095197 17230016

[B58] Javanmard M. Shlik J. Kennedy S. H. Vaccarino F. J. Houle S. Bradwejn J. (1999). Neuroanatomic correlates of CCK-4-induced panic attacks in healthy humans: a comparison of two time points. Biol. Psychiatry 45, 872–882. 10.1016/S0006-3223(98)00348-5 10202575

[B59] Jesuthasan S. (2012). Fear, anxiety, and control in the zebrafish. Dev. Neurobiol. 72, 395–403. 10.1002/dneu.20873 22328274

[B60] Jones E. G. Burton H. (1976). Projection from medial pulvinar to amygdala in primates. Brain Res. 104, 142–147. 10.1016/0006-8993(76)90654-5 813820

[B61] Kalueff A. V. Stewart A. M. Kyzar E. J. Cachat J. Gebhardt M. Landsman S. (2012). Time to recognize zebrafish ‘affective’ behavior. Behaviour 149, 1019–1036. 10.1163/1568539X-00003030

[B62] Karten H. J. (1969). Organization of avian telencephalon and some speculations on phylogeny of amniote telencephalon. Ann. N.Y. Acad. Sci. 167, 164–179. 10.1111/j.1749-6632.1969.tb20442.x

[B63] Kaas J. H. (2013). The evolution of the visual system in primates, in The New Visual Neurosciences, ed Werner J. Chalupa L. (Cambridge, MA: MIT Press), 1233–1246.

[B64] Kessler M. S. Debilly S. Schöppenthau S. Bielser T. Bruns A. Künnecke B.. (2012). fMRI fingerprint of unconditioned fear-like behavior in rats exposed to trimethylthiazoline. Eur. Neuropsychopharmacol. 22, 222–230. 10.1016/j.euroneuro.2011.07.011 21856130

[B65] Kozicz T. Lázár G. (1994). The origin of tectal npy immunopositive fibers in the frog. Brain Res. 635, 345–348. 10.1016/0006-8993(94)91460-5 8173975

[B66] Kozicz T. Lázár G. (2001). Colocalization of GABA, enkephalin and neuropeptide Y in the tectum of the green frog *Rana esculenta*. Peptides 22, 1071–1077. 10.1016/S0196-9781(01)00430-2 11445236

[B67] Kuraoka K. Konoike N. Nakamura K. (2015). Functional differences in face processing between the amygdala and ventrolateral prefrontal cortex in monkeys. Neurosci. 304, 71–80. 10.1016/j.neuroscience.2015.07.047 26208842

[B68] Kuraoka K. Nakamura K. (2006). Impacts of facial identity and type of emotion on responses of amygdala neurons. Neuroreport 17, 9–12. 10.1097/01.wnr.0000194383.02999.c5 16361941

[B69] Kuraoka K. Nakamura K. (2007). Responses of single neurons in monkey amygdala to facial and vocal emotions. J. Neurophysiol. 97, 1379–1387. 10.1152/jn.00464.2006 17182913

[B70] Laberge F. Mühlenbrock-Lenter S. Dicke U. Roth G. (2008). Thalamo-telencephalic pathways in the fire-bellied toad *Bombina orientalis*. J. Comp. Neurol. 508, 806–823. 10.1002/cne.21720 18395828

[B71] Laberge F. Roth G. (2007). Organization of the sensory input to the telencephalon in the fire-bellied toad, *Bombina orientalis*. J. Comp. Neurol. 502, 55–74. 10.1002/cne.21297 17335050

[B72] Ladu F. Bartolini T. Panitz S. G. Chiarotti F. Butail S. Macrì S.. (2015a). Live predators, robots, and computer-animated images elicit differential avoidance responses in zebrafish. Zebrafish 12, 205–214. 10.1089/zeb.2014.1041 25734228

[B73] Ladu F. Mwaffo V. Li J. Macrì S. Porfiri M. (2015b). Acute caffeine administration affects zebrafish response to a robotic stimulus. Behav. Brain Res. 289, 48–54. 10.1016/j.bbr.2015.04.020 25907748

[B74] Lázár G. (1989). Cellular architecture and connectivity of the frog's optic tectum and pretectum, in Visuomotor Coordination: Amphibians, Comparisons, Models, and Robots, ed Ewert J. P. Arbib M. A. (New York, NY: Plenum), 175–199.

[B76] Le Q. V. Isbell L. A. Matsumoto J. Le V. Q. Hori E. Tran A. H.. (2014). Monkey pulvinar neurons fire differentially to snake postures. PLoS ONE 9:e114258. 10.1371/journal.pone.0114258 25479158 PMC4257671

[B75] Le Q. V. Isbell L. A. Matsumoto J. Nguyen M. Hori E. Maior R. S.. (2013). Pulvinar neurons reveal neurobiological evidence of past selection for rapid detection of snakes. Proc. Natl. Acad. Sci. U.S.A. 110, 19000–19005. 10.1073/pnas.1312648110 24167268 PMC3839741

[B77] LeDoux J. E. (1990). The Emotional Brain. New York, NY: Simon & Schuster.

[B78] LeDoux J. E. (1994). Emotion, memory, and the brain. Sci. Am. 270, 50–57. 10.1038/scientificamerican0694-50 8023118

[B79] LeDoux J. E. (2000). Emotion circuits in the brain. Annu. Rev. Neurosci. 23, 155–184. 10.1146/annurev.neuro.23.1.155 10845062

[B80] Lent R. (1982). The organization of sub-cortical projections of the hamsters visual-cortex. J. Comp. Neurol. 206, 227–242. 10.1002/cne.902060303 7085930

[B81] Leonard C. M. Rolls E. T. Wilson F. A. Baylis G. C. (1985). Neurons in the amygdala of the monkey with responses selective for faces. Behav. Brain Res. 15, 159–176. 10.1016/0166-4328(85)90062-2 3994832

[B82] Liddell B. J. Brown K. J. Kemp A. H. Barton M. J. Das P. Peduto A.. (2005). A direct brainstem amygdala-cortical “alarm” system for subliminal signals of fear. Neuroimage 24, 235–243. 10.1016/j.neuroimage.2004.08.016 15588615

[B83] Lim L. W. Blokland A. Van Duinen M. Visser-Vandewalle V. Tan S. Vlamings R.. (2011). Increased plasma corticosterone levels after periaqueductal gray stimulation-induced escape reaction or panic attacks in rats. Behav. Brain Res. 218, 301–307. 10.1016/j.bbr.2010.12.026 21185871

[B84] Lima S. L. (1998). Stress and decision making under the risk of predation: recent developments from behavioral, reproductive, and ecological perspectives. Stress Behav. 27, 215–290. 10.1016/S0065-3454(08)60366-6

[B85] Lindqvist N. Näpänkangas U. Lindblom J. Hallböök F. (2003). Proopiomelanocortin and melanocortin receptors in the adult rat retinotectal system and their regulation after optic nerve transection. Eur. J. Pharmacol. 482, 85–94. 10.1016/j.ejphar.2003.10.011 14660008

[B86] Luca R. M. Gerlai R. (2012a). Animated bird silhouette above the tank: acute alcohol diminishes fear responses in zebrafish. Behav. Brain Res. 229, 194–201. 10.1016/j.bbr.2012.01.021 22266470 PMC3293988

[B87] Luca R. M. Gerlai R. (2012b). In search of optimal fear inducing stimuli: differential behavioral responses to computer animated images in zebrafish. Behav. Brain Res. 226, 66–76. 10.1016/j.bbr.2011.09.001 21920389 PMC3203217

[B88] Luiten P. G. M. (1981). Afferent and efferent connections of the optic tectum in the carp (*Cyprinus carpio* L). Brain Res. 220, 51–65. 10.1016/0006-8993(81)90210-9 6168333

[B89] Maior R. S. Hori E. Barros M. Teixeira D. S. Tavares M. C. H. Ono T.. (2011). Superior colliculus lesions impair threat responsiveness in infant capuchin monkeys. Neurosci. Lett. 504, 257–260. 10.1016/j.neulet.2011.09.042 21970966

[B90] Maior R. S. Hori E. Uribe C. E. Saletti P. G. Ono T. Nishijo H.. (2012). A role for the superior colliculus in the modulation of threat responsiveness in primates: toward the ontogenesis of the social brain. Rev. Neurosci. 23, 697–706. 10.1515/revneuro-2012-0055 23001312

[B91] Maximino C. Benzecry R. Matos Oliveira K. R. Oliveira Batista E. D. J. Herculano A. M. Rosemberg D. B. (2012). A comparison of the light/dark and novel tank tests in zebrafish. Behaviour 149, 1099–1123. 10.1163/1568539X-00003029

[B92] Maximino C. de Brito T. M. Colmanetti R. Assis Pontes A. A. de Castro H. M. de Lacerda R. I. T.. (2010a). Parametric analyses of anxiety in zebrafish scototaxis. Behav. Brain Res. 210, 1–7. 10.1016/j.bbr.2010.01.031 20117146

[B93] Maximino C. de Brito T. M. da Silva Batista A. W. Herculano A. M. Morato S. Gouveia A. Jr.. (2010b). Measuring anxiety in zebrafish: a critical review. Behav. Brain Res. 214, 157–171. 10.1016/j.bbr.2010.05.031 20510300

[B94] Maximino C. Lima M. G. Matos Oliveira K. R. Oliveira Batista E. D. J. Herculano A. M. (2013). “Limbic associative” and “autonomic” amygdala in teleosts: a review of the evidence. J. Chem. Neuroanat. 48–49, 1–13. 10.1016/j.jchemneu.2012.10.001 23137816

[B95] Montgomery N. M. Fite K. V. (1991). Organization of ascending projections from the optic tectum and mesencephalic pretectal gray in *Rana pipiens*. Vis. Neurosci. 7, 459–478. 10.1017/S0952523800009755 1764416

[B96] Moreno N. González A. (2005). Central amygdala in anuran amphibians: neurochemical organization and connectivity. J. Comp. Neurol. 489, 69–91. 10.1002/cne.20611 15977165

[B97] Moreno N. González A. (2006). The common organization of the amygdaloid complex in tetrapods: new concepts based on developmental, hodological and neurochemical data in anuran amphibians. Prog. Neurobiol. 78, 61–90. 10.1016/j.pneurobio.2005.12.005 16457938

[B98] Moreno N. González A. (2007). Evolution of the amygdaloid complex in vertebrates, with special reference to the anamnio-amniotic transition. J. Anat. 211, 151–163. 10.1111/j.1469-7580.2007.00780.x 17634058 PMC2375767

[B99] Morimoto N. Hashimoto K. Okada R. Mochida H. Uchiyama M. Kikuyama S.. (2011). Inhibitory effect of corticotropin-releasing factor on food intake in the bullfrog, *Aquarana catesbeiana*. Peptides 32, 1872–1875. 10.1016/j.peptides.2011.08.007 21864603

[B100] Morris J. S. deBonis M. Dolan R. J. (2002). Human amygdala responses to fearful eyes. Neuroimage 17, 214–222. 10.1006/nimg.2002.1220 12482078

[B101] Morris J. S. Frith C. D. Perrett D. I. Rowland D. Young A. W. Calder A. J.. (1996). A differential neural response in the human amygdala to fearful and happy facial expressions. Nature 383, 812–815. 10.1038/383812a0 8893004

[B102] Morris J. S. Ohman A. Dolan R. J. (1999). A subcortical pathway to the right amygdala mediating “unseen” fear. Proc. Natl. Acad. Sci. U.S.A. 96, 1680–1685. 10.1073/pnas.96.4.1680 9990084 PMC15559

[B103] Mueller T. (2012). What is the thalamus in zebrafish? Front. Neurosci. 6:64. 10.3389/fnins.2012.00064 22586363 PMC3345571

[B104] Mühlenbrock-Lenter S. Endepols H. Roth G. Walkowiak W. (2005). Immunohistological characterization of striatal and amygdalar structures in the telencephalon of the fire-bellied toad *Bombina orientalis*. Neuroscience 134, 705–719. 10.1016/j.neuroscience.2005.04.017 15961238

[B105] Müller-Ribeiro F. C. F. Dampney R. A. L. McMullan S. Fontes M. A. P. Goodchild A. K. (2014). Disinhibition of the midbrain colliculi unmasks coordinated autonomic, respiratory, and somatomotor responses to auditory and visual stimuli. Am. J. Physiol. Regul. Integr. Comp. Physiol. 307, R1025–R1035. 10.1152/ajpregu.00165.2014 25100075

[B106] Nakamura K. Mikami A. Kubota K. (1992). Activity of single neurons in the monkey amygdala during performance of a visual discrimination task. J. Neurophysiol. 67, 1447–1463. 1629757 10.1152/jn.1992.67.6.1447

[B107] Narayan E. J. Cockrem J. F. Hero J. M. (2013). Sight of a predator induces a corticosterone stress response and generates fear in an amphibian. PLoS ONE 8:e73564. 10.1371/journal.pone.0073564 24009756 PMC3757005

[B108] Northcutt R. G. Butler A. B. (1974). Retinal projections in northern water snake *Natrix sipedon sipedon* (L). J. Morphol. 142, 117–135. 10.1002/jmor.1051420202 4811244

[B109] Northcutt R. G. (2006). Connections of the lateral and medial divisions of the goldfish telencephalic pallium. J. Comp. Neurol. 494, 903–943. 10.1002/cne.20853 16385483

[B110] Northcutt R. G. (2008). Forebrain evolution in bony fishes. Brain Res. Bull. 75, 191–205. 10.1016/j.brainresbull.2007.10.058 18331871

[B111] Northcutt R. G. González A. (2011). A reinterpretation of the cytoarchitectonics of the telencephalon of the Comoran coelacanth. Front. Neuroanat. 5:9. 10.3389/fnana.2011.00009 21373374 PMC3046466

[B112] Northcutt R. G. Plassmann W. Holmes P. H. Saidel W. M. (2004). A pallial visual area in the telencephalon of the bony fish *Polypterus*. Brain Behav. Evol. 64, 1–10. 10.1159/000077538 15051962

[B113] Ogawa S. Nathan F. M. Parhar I. S. (2014). Habenular kisspeptin modulates fear in the zebrafish. Proc. Natl. Acad. Sci. U.S.A. 111, 3841–3846. 10.1073/pnas.1314184111 24567386 PMC3956168

[B114] Ohman A. Carlsson K. Lundqvist D. Ingvar M. (2007). On the unconscious subcortical origin of human fear. Physiol. Behav. 92, 180–185. 10.1016/j.physbeh.2007.05.057 17599366

[B115] Oliveira T. A. Koakoski G. Kreutz L. C. Ferreira D. Santos da Rosa J. G. de Abreu M. S.. (2013). Alcohol impairs predation risk response and communication in zebrafish. PLoS ONE 8:e75780. 10.1371/journal.pone.0075780 24116073 PMC3792133

[B116] Olsen C. M. Lovering A. T. Carr J. A. (1999). Alpha-melanocyte-stimulating hormone and habituation of prey-catching behavior in the Texas toad, *Bufo speciosus*. Horm. Behav. 36, 62–69. 10.1006/hbeh.1999.1531 10433887

[B117] Pasley B. N. Mayes L. C. Schultz R. T. (2004). Subcortical discrimination of unperceived objects during binocular rivalry. Neuron 42, 163–172. 10.1016/S0896-6273(04)00155-2 15066273

[B118] Pelosi G. G. Tavares R. F. Fernandes K. B. P. Corrêa F. M. A. (2009). Cardiovascular effects of noradrenaline microinjection into the medial part of the superior colliculus of unanesthetized rats. Brain Res. 1290, 21–27. 10.1016/j.brainres.2009.07.009 19615348

[B119] Pepels P. P. Meek J. Bonga S. E. W. Balm P. H. M. (2002). Distribution and quantification of corticotropin-releasing hormone (CRH) in the brain of the teleost fish *Oreochromis mossambicus* (tilapia). J. Comp. Neurol. 453, 247–268. 10.1002/cne.10377 12378586

[B120] Perusini J. N. Fanselow M. S. (2015). Neurobehavioral perspectives on the distinction between fear and anxiety. Learn. Mem. 22, 417–425. 10.1101/lm.039180.115 26286652 PMC4561408

[B121] Pessoa L. Adolphs R. (2010). Emotion processing and the amygdala: from a ‘low road’ to ‘many roads’ of evaluating biological significance. Nat. Rev. Neurosci. 11, 773–782. 10.1038/nrn2920 20959860 PMC3025529

[B122] Pettigrew J. D. Konishi M. (1976a). Effect of monocular deprivation on binocular neurons in owls visual wulst. Nature 264, 753–754. 10.1038/264753a0 1012314

[B123] Pettigrew J. D. Konishi M. (1976b). Neurons selective for orientation and binocular disparity in visual wulst of barn owl (*Tyto alba*). Science 193, 675–678. 10.1126/science.948741 948741

[B124] Prater C. M. Carr J. A. Garcia C. Harris B. (2014). Food deprivation and stressor exposure alter tectal CRF concentrations in African clawed frogs *Xenopus laevis*. Integr. Comp. Biol. 54, E333–E333.

[B125] Preuss S. J. Trivedi C. A. vom Berg-Maurer C. M. Ryu S. Bollmann J. H. (2014). Classification of object size in retinotectal microcircuits. Curr. Biol. 24, 2376–2385. 10.1016/j.cub.2014.09.012 25242030

[B126] Ratner S. C. (1967). Comparative aspects of hypnosis, in Handbook of Clinical and Experimental Hypnosis, ed Gordon J. E. (New York, NY: Macmillan), 550–587.

[B127] Rau V. DeCola J. P. Fanselow M. S. (2005). Stress-induced enhancement of fear learning: an animal model of posttraumatic stress disorder. Neurosci. Biobehav. Rev. 29, 1207–1223. 10.1016/j.neubiorev.2005.04.010 16095698

[B128] Rettig G. (1988). Connections of the tectum opticum in 2 urodeles, *Salamandra salamandra* and *Bolitoglossa subpalmata*, with special reference to the nucleus isthmi. J. Hirnforsch. 29, 5–16. 3385198

[B129] Rodrigues S. M. LeDoux J. E. Sapolsky R. M. (2009). The influence of stress hormones on fear circuitry. Annu. Rev. Neurosci. 32, 289–313. 10.1146/annurev.neuro.051508.135620 19400714

[B130] Romanski L. M. Giguere M. Bates J. F. Goldman-Rakic P. S. (1997). Topographic organization of medial pulvinar connections with the prefrontal cortex in the rhesus monkey. J. Comp. Neurol. 379, 313–332. 9067827

[B131] Roth G. Grunwald W. (2000). Morphology, axonal projection pattern, and responses to optic nerve stimulation of thalamic neurons in the salamander *Plethodon jordani*. J. Comp. Neurol. 428, 543–557. 10.1002/1096-9861(20001218)428:33.0.CO;2-X 11074450

[B132] Roth G. Grunwald W. Dicke U. (2003). Morphology, axonal projection pattern, and responses to optic nerve stimulation of thalamic neurons in the fire-bellied toad *Bombina orientalis*. J. Comp. Neurol. 461, 91–110. 10.1002/cne.10670 12722107

[B133] Schneider G. E. (1967). Contrasting visuomotor functions of tectum and cortex in the golden hamster. Psychol. Forsch. 31, 52–62. 10.1007/BF00422386 5605117

[B134] Schneider G. E. (1969). 2 visual systems. Science 163, 895–902. 10.1126/science.163.3870.895 5763873

[B135] Schwippert W. W. Ewert J. P. (1995). Effect of neuropeptide-Y on tectal field potentials in the toad. Brain Res. 669, 150–152. 10.1016/0006-8993(94)01260-O 7712160

[B136] Schwippert W. W. Rottgen A. Ewert J. P. (1998). Neuropeptide Y (NPY) or fragment NPY 13-36, but not NPY 18-36, inhibit retinotectal transfer in cane toads *Bufo marinus*. Neurosci. Lett. 253, 33–36. 10.1016/S0304-3940(98)00596-5 9754798

[B137] Shahbazi M. Schmidt M. Carruth L. L. (2011). Distribution and subcellular localization of glucocorticoid receptor-immunoreactive neurons in the developing and adult male zebra finch brain. Gen. Comp. Endocrinol. 174, 354–361. 10.1016/j.ygcen.2011.09.017 21986090

[B138] Shang C. P. Liu Z. H. Chen Z. J. Shi Y. C. Wang Q. Liu S.. (2015). A parvalbumin-positive excitatory visual pathway to trigger fear responses in mice. Science 348, 1472–1477. 10.1126/science.aaa8694 26113723

[B139] Sharma S. C. (1975). Visual projection in surgically created compound tectum in adult goldfish. Brain Res. 93, 497–501. 10.1016/0006-8993(75)90188-2 1174982

[B140] Shibasaki M. Nagumo S. Koda H. (2014). Japanese monkeys (*Macaca fuscata*) spontaneously associate alarm calls with snakes appearing in the left visual field. J. Comp. Psychol. 128, 332–335. 10.1037/a0036049 24611644

[B141] Shimizu T. Bowers A. N. (1999). Visual circuits of the avian telencephalon: evolutionary implications. Behav. Brain Res. 98, 183–191. 10.1016/S0166-4328(98)00083-7 10683106

[B142] Shoukfeh O. M. Ahmed N. Carr J. A. (2003). Neurochemical coding of a behavioral circuit breaker. Integr. Comp. Biol. 43, 882–882.

[B143] Silva P. I. Martins C. I. Khan U. W. Gjøen H. M. Øverli Ø. Höglund E. (2015). Stress and fear responses in the teleost pallium. Physiol. Behav. 141, 17–22. 10.1016/j.physbeh.2014.12.020 25497079

[B144] Simpson G. G. (1961). Principles of Animal Taxonomy. New York, NY: Columbia University Press. 10.1126/science.133.3464.1589 17781120

[B145] Stepniewska W. (2004). The pulvinar complex, in Primate Visual Systems, eds Kaas J. H. Collins C. E. (Boca Raton, FL: CRC Press), 53–80.

[B146] Steuwe C. Daniels J. K. Frewen P. A. Densmore M. Theberge J. Lanius R. A. (2015). Effect of direct eye contact in women with PTSD related to interpersonal trauma: psychophysiological interaction analysis of connectivity of an innate alarm system. Psychiatry Res. 232, 162–167. 10.1016/j.pscychresns.2015.02.010 25862529

[B147] Striedter G. F. (1990). The diencephalon of the channel catfish, *Ictalurus punctatus*.2. Retinal, tectal, cerebellar and telencephalic connections. Brain Behav. Evol. 36, 355–377. 10.1159/000115319 2073574

[B148] Tamietto M. de Gelder B. (2010). Neural bases of the non-conscious perception of emotional signals. Nat. Rev. Neurosci. 11, 697–709. 10.1038/nrn2889 20811475

[B149] Teitsma C. A. Anglade I. Toutirais G. Muñoz-Cueto J. A. Saligaut D. Ducouret B.. (1998). Immunohistochemical localization of glucocorticoid receptors in the forebrain of the rainbow trout (*Oncorhynchus mykiss*). J. Comp. Neurol. 401, 395–410. 9811116

[B150] Temizer I. Donovan J. C. Baier H. Semmelhack J. L. (2015). A visual pathway for looming-evoked escape in larval zebrafish. Curr. Biol. 25, 1823–1834. 10.1016/j.cub.2015.06.002 26119746

[B151] Toledo L. F. Sazima I. Haddad C. F. B. (2010). Is it all death feigning? Case in anurans. J. Nat. His. 44, 1979–1988. 10.1080/00222931003624804

[B152] van Groen T. Wyss J. M. (1992). Projections from the laterodorsal nucleus of the thalamus to the limbic and visual cortices in the rat. J. Comp. Neurol. 324, 427–448. 10.1002/cne.903240310 1383292

[B153] Venkatesan S. Carr J. A. (2001). Distribution of neuronal melanocortins in the spadefoot toad *Spea multiplicata* and effects of stress. Brain Behav. Evol. 57, 150–160. 10.1159/000047233 11509823

[B154] Verbeek P. Iwamoto T. Murakami N. (2008). Variable stress-responsiveness in wild type and domesticated fighting fish. Physiol. Behav. 93, 83–88. 10.1016/j.physbeh.2007.08.008 17884114

[B155] Volchan E. Souza G. G. Franklin C. M. Norte C. E. Rocha-Rego V. Oliveira J. M.. (2011). Is there tonic immobility in humans? Biological evidence from victims of traumatic stress. Biol. Psychol. 88, 13–19. 10.1016/j.biopsycho.2011.06.002 21693167

[B156] von Wietersheim A. Ewert J. -P. (1978). Neurons of the toad's (*Bufo bufo* L.) visual system sensitive to moving configurational stimuli: a statistical analysis. J. Comp. Physiol. 126, 35–42. 10.1007/BF01342648

[B157] Wei P. Liu N. Zhang Z. Liu X. Tang Y. He X.. (2015). Processing of visually evoked innate fear by a non-canonical thalamic pathway. Nat. Comm. 6:6756. 10.1038/ncomms7756 25854147 PMC4403372

[B158] Weller R. E. Kaas J. H. (1989). Parameters affecting the loss of ganglion-cells of the retina following ablations of striate cortex in primates. Vis. Neurosci. 3, 327–349. 10.1017/S0952523800005514 2487111

[B159] Werner E. E. Anholt B. R. (1993). Ecological consequences of the trade-off between growth and mortality-rates mediated by foraging activity. Am. Nat. 142, 242–272. 10.1086/285537 19425978

[B160] Wiggert B. O. Chader G. J. (1975). Glucocorticoid and progesterone receptor in chick optic tectum. J. Neurochem. 24, 585–586. 10.1111/j.1471-4159.1975.tb07679.x 163297

[B161] Wilczyniski W. Northcutt R. G. (1977). Afferents to the optic tectum of the leopard frog: an HRP study. J. Comp. Neurol. 173, 219–230 10.1002/cne.901730202 300743

[B162] Woodley C. M. Peterson M. S. (2003). Measuring responses to simulated predation threat using behavioral and physiological metrics: the role of aquatic vegetation. Oecologia 136, 155–160. 10.1007/s00442-003-1236-1 12684858

[B163] Yamamoto N. Ito H. (2008). Visual, lateral line, and auditory ascending pathways to the dorsal telencephalic area through the rostrolateral region of the lateral preglomerular nucleus in cyprinids. J. Comp. Neurol. 508, 615–647. 10.1002/cne.21717 18381599

[B164] Yao M. Hu F. Denver R. J. (2008). Distribution and corticosteroid regulation of glucocorticoid receptor in the brain of *Xenopus laevis*. J. Comp. Neurol. 508, 967–982. 10.1002/cne.21716 18399546

